# Association between Socioeconomic Position of the Family and Adolescent Obesity in Germany—Analysis of the Mediating Role of Familial Determinants

**DOI:** 10.1155/2024/7903972

**Published:** 2024-11-05

**Authors:** Miriam Blume, Anja Schienkiewitz, Lina Wollgast, Stephanie Hoffmann, Lydia Sander, Jacob Spallek, Raphael M. Herr, Irene Moor, Claudia R. Pischke, Iryna Iashchenko, Claudia Hövener, Petra Rattay

**Affiliations:** ^1^Department of Epidemiology and Health Monitoring, Robert Koch Institute, Berlin, Germany; ^2^Department of Public Health, Brandenburg University of Technology Cottbus-Senftenberg, Senftenberg, Germany; ^3^Lusatian Center for Digital Public Health, Brandenburg University of Technology Cottbus-Senftenberg, Senftenberg, Germany; ^4^Center for Preventive Medicine and Digital Health, Medical Faculty Mannheim, Heidelberg University, Heidelberg, Germany; ^5^Department of Medical Informatics, Biometry and Epidemiology, Friedrich–Alexander–Universität Erlangen–Nürnberg, Erlangen, Germany; ^6^Institute of Medical Sociology, Interdisciplinary Center for Health Sciences, Medical Faculty, Martin–Luther–University Halle-Wittenberg, Halle, Germany; ^7^Institute of Medical Sociology, Centre for Health and Society, Medical Faculty, Heinrich–Heine–University Duesseldorf, Duesseldorf, Germany; ^8^Chair of Health Economics, School of Medicine and Health, Technical University of Munich, Munich, Germany

## Abstract

**Background:**

Obesity's negative impact on young people's health has long been known. The family and its socioeconomic position (SEP) are key determinants in adolescent obesity. However, understanding which familial determinants explain the association remains limited.

**Method:**

The analyses are based on data from the “German Health Interview and Examination Survey for Children and Adolescents” (KiGGS) (1,384 females and 1,332 males aged 11 to 17 years). Logistic regression models explored how familial determinants (family stress, family cohesion, parental smoking, parental sporting activity, and parental overweight) mediated the association between family SEP (parental education, occupational status, and household income) and adolescent obesity.

**Results:**

Significant total effects for the associations between family SEP in childhood and adolescent obesity were found. Splitting the total effect of the family SEP on obesity into direct and indirect effects, all direct effects turned out to be significant. However, all associations involved also indirect effects of familial determinants, except for household income for female adolescents. Parental smoking and overweight were the most relevant mediators for males and females. For male adolescents, parental sporting activity additionally mediated the association between SEP and obesity.

**Conclusion:**

A low SEP in childhood was associated with adolescent obesity. Parental health and health behaviors partly explained the association. For increasing health equality in adolescent health, the consideration of parental health behavior in the planning and implementation of health promotion programs seem to be important.

## 1. Introduction

Young people's obesity is a public health concern in many countries worldwide [1]. In Germany, 6.3% of 11- to 13-year-olds and 5.9% of 14- to 17-year-olds female adolescents were obese in 2018 according to the WHO reference system. Among male adolescents, 12.0% and 9.5% of the 11- to 13-year-olds and 14- to 17-year-olds were obese, respectively [2].

Obesity during childhood and adolescence increases the risk for developing short- and long-term health consequences. These increase the risk for morbidity, including a higher risk of developing cancer, cardiovascular disease, metabolic syndrome, and type 2 diabetes as well as an earlier mortality risk in later life [3]. For every two years that an individual is obese, the risk for mortality increases by 6 to 7% [4].

The family SEP is strongly associated with the health of adolescents [5]. Accordingly, studies have shown that socioeconomically disadvantaged adolescents have a higher probability of obesity than adolescents from families with a higher SEP [6, 7]. During the last decades, an increase of socioeconomic inequalities in obesity in high-income countries was observed. From 1988 to 2011, the prevalence of obesity among adolescents of middle and high SEP groups stagnated, whereas an increase in obesity prevalence among low SEP adolescents was observed during the same time period [6].

Obesity is affected by a complex interplay of different individual, societal, and environmental factors. Especially, the family is important in the investigation of obesity [8]. Many studies identified the family environment as a significant determinant influencing the health and weight of children [8–10]. For example, family communication, behavior control, family cohesion as well as family conflict are associated with adolescent obesity [9]. A meta-analysis found that children are 1.97 times more likely to be overweight or obese if their parents are also overweight or obese [10].

Some studies found that familial determinants mediate the association between SEP and adolescent obesity, which means that the association can be partly or fully explained by family determinants. For example, parental weight and shared family meals were found to be important in explaining the socioeconomic differences in obesity [11–13]. However, studies often focus on younger children rather than older children and adolescents. Moreover, previous work often analyzed the total mediating effect of all familial determinants and therefore could not differentiate between the individual effect of different familial determinants.

The aim of this analysis is to add to the current knowledge by exploring the association between family SEP in childhood and obesity in adolescents as well as the role of different familial determinants in explaining health inequalities. The following research questions were addressed:

1. Is the family SEP in childhood associated with obesity in male and female adolescents in Germany (total effect)?
2. Do familial determinants mediate the association between family SEP and adolescent obesity? How strong are the direct and indirect effects of the family SEP on obesity?
3. Which familial determinants are particularly relevant for explaining the association between family SEP in childhood and obesity in male and female adolescents?

## 2. Materials and Methods

### 2.1. Data

The analyses were carried out using cohort data from the “German Health Interview and Examination Survey for Children and Adolescents” (KiGGS) conducted by the Robert Koch Institute. The survey included physical examinations and interviews. KiGGS baseline (t0) was carried out between 2003 and 2006 and included children and adolescents up to the age of 17 years. The first follow-up study, KiGGS Wave 1 (t1), took place between 2009 and 2012 and included 6- to 26-year-old study participants. Parent- and self-reported information was collected through telephone interviews. KiGGS Wave 2 (t2) (2014–2017) included 10- to 31-year-old participants and was carried out as a health interview and examination survey similar to the baseline study. Further details about the study design can be found in the article by Mauz et al. [14].

**Figure 1:**
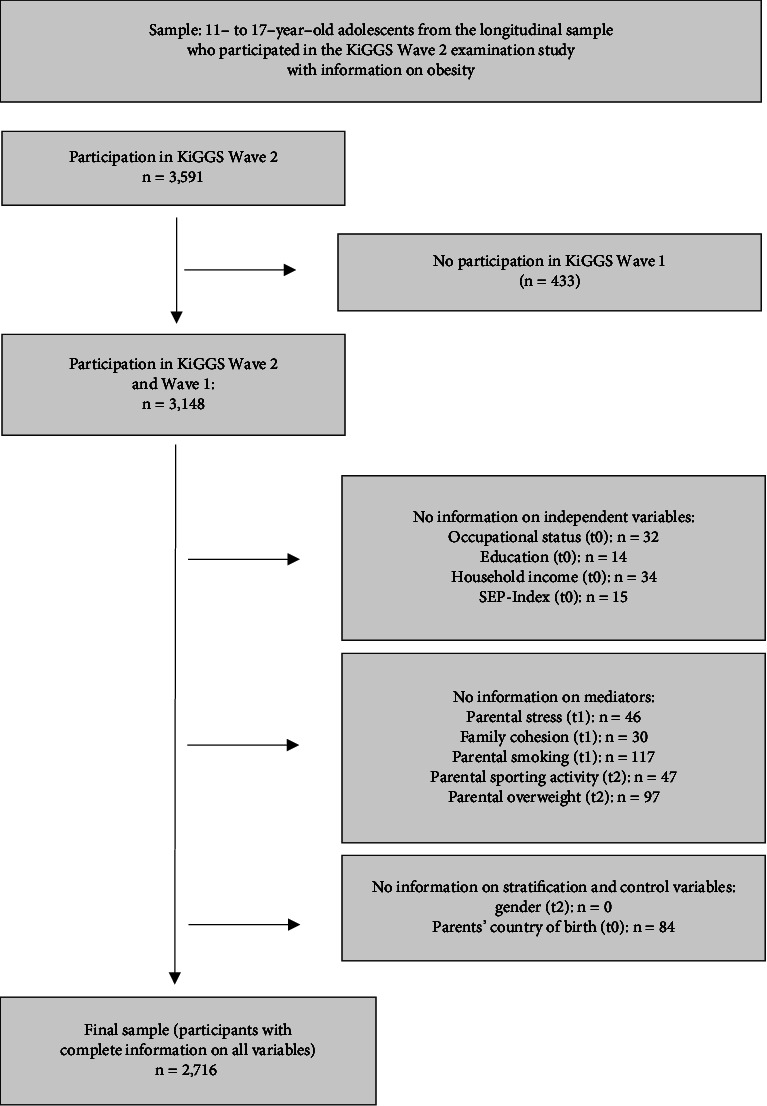
Flowchart on sample size and missing values.

In the KiGGS cohort, we have information on obesity for 3,591 11- to 17-year-olds in Wave 2. Of these, 3,149 also participated in KiGGS Wave 1. Participants for whom complete information on all variables was not available (*n* = 433) were excluded from the analysis (see Figure 1), resulting in a sample of 1,332 male and 1,384 female adolescents aged 11 to 17 years at the time of Wave 2 (see Figure 1). The mean age was 14.05 (SD 2.00) years. The sample characteristics are shown in Table 1.

### 2.2. Variables

#### 2.2.1. Outcome Variable

Based on measured weight and height, obesity was defined as a body mass index (kg/m^2^) above the 97^th^ age- and sex-specific percentile using the national reference system by Kromeyer–Hauschild et al. [15, 16]. For the present analyses, the outcome variable obesity at Wave 2 was dichotomized (yes/no).

#### 2.2.2. Independent Variables

The independent variables parental education level, parental occupational status, household income, and the SEP index based on these three variables were used from KiGGS baseline. Level of parental education was measured using the educational classification of the “Comparative Analysis of Social Mobility in Industrial Nations” (CASMIN) [17]. It considers the graduation and occupational qualifications and is standardized into a score with a range from one to seven, with one being the lowest educational status. Occupational status of the parents was determined via the “International Socio–Economic Index of Occupational Status” (ISEI) by Ganzeboom et al. [18]. The score ranges from one to seven, with one indicating the lowest occupational status. Household income was determined through the net equivalent income of the household. The net equivalent income is calculated based on the income of the household and the number of people living in the household, considering each person's age. In the case of categorical or missing information, the information was being distributed evenly on the equivalent interval or imputed [19]. The net equivalent income was summarized into values between one and seven, with one being the lowest income status. The multidimensional index of socioeconomic position (SEP index) was created by adding the scores of education, household income, and occupational status. The sum was then divided by three, resulting in a range of one (low SEP) to seven (high SEP) [20].

#### 2.2.3. Mediating Variables

Five familial determinants were considered as mediating variables. In the case of single-parent families, only the values of one parent were included. Parental smoking was determined by the question “Do you currently smoke?” and “Does your partner currently smoke?”, defined by at least one parent smoking (yes/no). The response categories “yes, daily,” and “yes, occasionally” were combined. Parental overweight was defined as at least one parent being overweight or obese (yes/no), with overweight defined by a BMI ≥25, according to the WHO recommendation [21]. Parental sporting activity during the last three months was captured by the question “How often do you exercise?” and the parameter values “no sporting activity”, “<1 hour a week”, “regularly, 1–2 hours a week”, “regularly, 2–4 hours a week”, “regularly, >4 hours a week.” The values for both parents were added up and divided by two, resulting in a range from zero to five. Parental stress captured thirteen different potential stressors, which included burden by household work, financial worries, sole responsibility for parenting, family members in need of care, parenting problems or conflicts, conflicts with an (ex-)partner or other family members, loneliness, occupational situation or unemployment, lack of recognition of household and family chores, a disabled or chronically ill child, and conflicts of compatibility of family and work. Response choices were captured on a five-point scale ranging from “not at all” to “very much” [22]. The potential stressors were added up and then divided by 13. All four familial determinants mentioned above were answered by the parents. Family cohesion is an instrument based on four variables, developed by Schneewind et al. [23], and was answered by adolescents themselves. Answers were given to the following statements: “In our family, everyone responds to the concerns and needs of the other.”; “We really all get along well.”; “We are enthusiastic about everything we do at home.”; and “In our family, everyone feels like they are being listened to and responded to.” Answers to the questions were given on a four-point scale, ranging from 1 “disagree” to 4 “agree,” which were then summed up and converted to an index ranging from zero to 100.

#### 2.2.4. Control Variables

Two control variables were included in the logistic regression models. The country of birth of the parents was categorized into “both parents born in Germany,” “both parents not born in Germany,” and “one parent born in Germany.” Age was included as a continuous variable with whole years from Wave 2.

### 2.3. Statistical Analyses

We performed weighted logistic regression models employing the method by Karlson, Holm, and Breen [24, 25] to decompose the total effects of the SEP variables on obesity into the direct effects of these predictors and the indirect effects through familial determinants (Table 2). The KHB method allows for comparing the estimated coefficients of two nested nonlinear probability models. It is a general decomposition method that is unaffected by rescaling or attenuation bias that arises in cross-model comparisons in nonlinear models [25].

The KHB method also allows to quantify the degree to which all familial determinants mediate the association between the SEP variables and obesity. Therefore, the overall mediation percentage (by Karlson, Holm, and Breen referred to as “confounding percentage” [25]) for all mediators together is reported in Table 2. In the next step, the respective explanatory percentage for each mediator variable is displayed separately (Table 3) [24, 25].

As the individual SEP variables correlate with each other (see appendix, Supplementary Table A1), a separate model was calculated for each SEP indicator. Instead of including income, education, and occupational status simultaneously in one model, the SEP index was used. All analyses were performed stratified by gender.

Additionally, in preparation for the mediation analysis, point-biserial correlation coefficients were calculated to analyze whether each SEP variable was associated with obesity in male and female adolescents separately (see appendix, Supplementary Table A2). The point-biserial correlation is appropriate for associations between metric exposures and categorical outcomes. Additionally, correlation coefficients between all mediating variables were calculated to test for multicollinearity.

The data were weighted based on age, sex, region, nationality, and the SEP of the family in KiGGS baseline. As a cohort sample was analyzed, a longitudinal weighting factor was used in the statistical analyses to compensate for biases in the sample due to selective reparticipation and to account for the clustered sample design. This created a weighted sample based on KiGGS baseline [26].

The analyses were carried out using Stata (version 17.0) software. Differences were considered statistically significant when *p* values were lower than 0.05.

## 3. Results

For all SEP variables at baseline (education, occupational status, household income, and SEP index), we found significant total effects on obesity at Wave 2, showing that a low SEP in childhood is associated with obesity in adolescence. This was the case for female and male adolescents (Table 2).

For all SEP indicators, we observed significant direct effects on obesity (Table 2). The indirect effects were significant for all SEP indicators except for household income in female adolescents (Table 2). Thus, all the associations between family SEP and obesity—except for income in female adolescents—were partly mediated by familial determinants. The mediation percentages of familial determinants differed across individual SEP variables. All familial determinants combined explained 36.8%, 29.6%, 26.7%, and 28.0% of the total effects of household income, education, occupational status, and the SEP index on obesity among male adolescents. Among female adolescents, the total mediation percentages of familial determinants explaining the association of education, occupational status, and the SEP index with obesity were 32.5%, 23.5%, and 19.6%, respectively. For the association between household income and obesity, the total mediation percentage of all familial determinants was 9.7% (not significant) in female adolescents (see Table 2).

In the next step, we investigated in detail which familial determinants explain the association of childhood SEP and adolescent obesity (see Table 3). We found that parental sporting activity, parental smoking, and parental overweight were relevant mediators for male adolescents. Among female adolescents, parental smoking and parental overweight were important mediators of the association between family SEP and obesity. Since the indirect effect of household income on female adolescent obesity was not significant, we reported no mediation percentage as shown in Table 3.

## 4. Discussion

The aim of this study was to analyze the association between childhood SEP and obesity among adolescents, as well as the mediating role of the family. We found significant total effects of all socioeconomic determinants on female and male adolescent obesity. Furthermore, all direct effects of the SEP determinants on obesity turned out to be significant. This means that there are relevant differences in obesity depending on the familial SEP, which remained stable even when familial determinants were considered which is in line with previous studies that also described an association between SEP and obesity [6]. Furthermore, we found that familial determinants partly mediate the association between childhood family SEP and adolescent obesity. Focusing on the indirect pathways, we observed for all SEP indicators significant indirect effects on adolescent obesity through family determinants, except for the association between income and obesity in female adolescents.

The mediation analyses revealed that the relevant familial determinants are all related to parental health behavior (parental overweight, parental sporting activity, and parental smoking). In a study by Bammann et al. [7], familial determinants (e.g., feeding/eating practices, parental body mass index, physical activity behavior, and proportion of sedentary activity) also partly explained the association between SEP and obesity. Other studies have shown that parental health behavior influences adolescent health behavior and, as a result, their weight [27]. The finding that parental weight was an important mediator regarding the association between SEP and adolescent obesity is consistent with other studies [11–13]. Bandura's social-cognitive theory supports the results, as it addresses the extent to which the parental health behavior has an influence on children's health [28]. Bandura [28] describes the family as a central institution for the development of one's own competence in health and health behavior. Growing up, children learn in the familial context by observing social role models performing health behavior. Hence, health and health behavior are shaped early in the family and often continue throughout the life course [28].

In our analyses, family cohesion and family stress did not mediate the association between SEP and obesity. This is contradictory to the family stress model which points out that poverty or financial hardship can cause parental stress and family conflicts and in consequence has a negative effect on children and adolescents [29]. Studies applying the family stress model more often focused on mental health outcomes than obesity [30]. The model by Hemmingsson [31] focuses on the association between SEP and overweight/obesity in childhood and adolescence specifically. In this model, psychosocial factors of the parents and offspring are taken into account. Although low SEP is associated with increased parental stress, which may affect the family environment and offspring stress [31], the children's stress itself appears to be a more important risk factor for obesity than their parents' stress [32]. Similar to this study, we also did not see a mediation effect of parental stress.

## 5. Strengths and Limitations

The main strength of our analysis is the large study sample with data from three population-based survey waves of KiGGS. Another advantage is that we used measurement data on height and weight, which are more valid than self-reported data [33]. Additionally, the data included information on several socioeconomic and familial determinants. Although the predictors (family SEP) were collected prior to the familial mediators and the outcome (obesity), we cannot draw any conclusions about a causal direction because we did not control for obesity or familial determinants in earlier life years (childhood). Also, there are periods of almost six years between the survey waves, in which the family mediator variables do not have to be stable over time. In the study, we cannot represent the family situation over the entire time period. Furthermore, some mediator variables were only collected in KiGGS Wave 2 and therefore could not be included from Wave 1. As in most cohort studies, there is nonrandom dropout, particularly among adolescents from families with low SEP. Despite the application of a weighting factor to account for this attrition, the effect of SEP on obesity may be underestimated in the present study.

## 6. Conclusion

It can be concluded that the family SEP in childhood as well as parental health behavior plays an important role in adolescent obesity. Thus, the family represents a fundamental determinant and setting for adolescent health [34]. Especially parents' health behavior seems to be a key factor in preventing adolescent obesity. Promoting parents' and children's health behavior may help reduce obesity among young people, especially with a low SEP. In order to promote a healthy behavior, the World Health Organization [35] advocates for a balance of target group-specific interventions on the one hand and population-wide approaches on the other hand to prevent obesity and to reduce the incidence of obesity. Population-wide interventions that may address, for instance, food labelling, pricing, and availability should be complemented by community-level interventions with a focus on particularly vulnerable groups [35]. In order to comprehensively address health inequalities in adolescent obesity interventions based on the family environment, other relevant settings such as schools (mesolevel) and the macrolevel are crucial.

## Figures and Tables

**Table 1:** Sample characteristics for 11- to 17-year-old adolescents.

	Males (*n* = 1.332)		Females (*n* = 1.384)	
*Wave 2 (t2)*				
Obesity yes (%, [95% CI])	8.5	[6.5–11.0]	7.6	[5.5–10.4]
Parental overweight yes (%, [95% CI])	71.7	[68.3–74.9]	70.6	[67.3–73.7]
Parental sporting activity (1–5) (mean, SD)	2.56	1.12	2.57	1.11
Age (11–17) (mean, SD)	14.10	1.98	14.09	1.98

*Wave 1 (t1)*				
Parental smoking yes (%, [95% CI])	44.9	[41.2–48.6]	42.36	[38.5–46.3]
Family cohesion (0–100) (mean, SD)	79.30	13.23	79.84	13.66
Parental stress (1–5) (mean, SD)	1.75	0.50	1.71	0.50

*Baseline (t0)*				
Education (1–7) (mean, SD)	4.36	1.51	4.45	1.53
Occupational status (1–7) (mean, SD)	3.16	1.32	3.25	1.31
Household income (1–7) (mean, SD)	4.11	1.78	4.22	1.77
SEP–index (1–7) (mean, SD)	3.87	1.28	3.97	1.24

% (weighted prevalence); 95% CI (weighted); mean (weighted); SD (weighted).

**Table 2:** Decomposition of the total effects of the SEP indicators on obesity by familial determinants for female and male adolescents.

	Males (*n* = 1,332)				Females (*n* = 1,384)			
	OR	95% CI		*p*	OR	95% CI		*p*
*Education*								
Total effect	1.65	1.23	2.21	0.001	1.61	1.26	2.05	<0.001
Direct effect	1.42	1.05	1.93	0.025	1.38	1.09	1.75	0.008
Indirect effect	1.16	1.05	1.28	0.004	1.17	1.06	1.29	0.003
*R* ^2^ (full model)	0.14				0.14			
Mediation %	29.6%				32.5%			

*Occupational status*								
Total effect	1.60	1.19	2.14	0.002	1.71	1.20	2.44	0.003
Direct effect	1.41	1.04	1.91	0.028	1.51	1.06	2.15	0.023
Indirect effect	1.13	1.02	1.26	0.016	1.13	1.03	1.25	0.014
*R* ^2^ (full model)	0.13				0.15			
Mediation %	26.7%				23.5%			

*Household income*								
Total effect	1.37	1.14	1.65	0.001	1.44	1.19	1.74	<0.001
Direct effect	1.22	1.00	1.48	0.047	1.39	1.16	1.66	<0.001
Indirect effect	1.12	1.03	1.23	0.010	1.04	0.95	1.12	0.401
*R* ^2^ (full model)	0.13				0.15			
Mediation %	36.8%				9.7%			

*SEP index*								
Total effect	1.89	1.38	2.59	<0.001	2.13	1.52	2.99	<0.001
Direct effect	1.58	1.12	2.23	0.009	1.84	1.33	2.53	<0.001
Indirect effect	1.20	1.04	1.37	0.010	1.16	1.01	1.33	0.030
*R* ^2^ (full model)	0.14				0.16			
Mediation %	28.0%				19.6%			

*Note.* All mediators are used simultaneously in all models. All models are adjusted for age and country of birth of the parents.

**Table 3:** Proportions (in %) of the indirect effects of each familial determinant in explaining the association between SEP and obesity in female and male adolescents.

Mediators	Education %	Occupational status (%)	Household income (%)	SEP index (%)
*Males*				
Parental sporting activity	**16.8**	**25.1**	**23.4**	**18.9**
Parental smoking	**32.8**	**38.0**	**39.7**	**34.3**
Parental overweight	**49.8**	**41.4**	**31.9**	**44.9**
Parental stress	−1.7	−5.7	4.3	0.4
Family cohesion	2.3	1.2	0.8	1.6

*Females*				
Parental sporting activity	5.8	8.0	—	4.5
Parental smoking	**32.7**	**39.7**	—	**47.7**
Parental overweight	**55.6**	**52.7**	—	**44.7**
Parental stress	3.8	6.2	—	0.8
Family cohesion	2.1	−6.6	—	2.3

*Note.* Proportions >10% are printed in bold and considered relevant; —: no significant indirect effect.

## Data Availability

For ethical and legal reasons, data from the KiGGS study are only available on request. The informed consent of the study participants did not cover the public deposition of data. However, the minimal dataset underlying the findings is archived in the “Health Monitoring” Research Data Centre at the Robert Koch Institute (RKI) and can be accessed by all interested researchers on-site. The “Health Monitoring” Research Data Centre is accredited by the German Data Forum according to uniform and transparent standards (https://www.ratswd.de/en/datainfrastructure/rdc). On-site access to the minimal data set is possible at the Secure Data Centre of the RKI's “Health Monitoring” Research Data Centre. Requests should be addressed to Dr Ronny Kuhnert at the Robert Koch Institute, “Health Monitoring” Research Data Centre, General-Pape-Straße 64, 12101 Berlin, Germany (e-mail: fdz@rki.de).

## Abbreviations

SEP: Socioeconomic position
SD: Standard deviation
CI: Confidence interval
BMI: Body mass index
KHB method: Statistical method by Karlson, Holm and Breen
OR: Odds ratio
p: *p* value
Coef: Coefficient.

## Supplementary Materials

Supplementary Materials Table A1: Pearson's Correlation Coefficient for the associations between the single SEP variables (*n* = 2,716). Table A2: point-biserial correlation coefficient for the association with adolescent's obesity.
