# Supplementary material for: Association between Socioeconomic Position of the Family and Adolescent Obesity in Germany—Analysis of the Mediating Role of Familial Determinants
Source: J Obes. 2024 Nov 5;2024:7903972. doi: 10.1155/2024/7903972 (PMC11557177; doi:10.1155/2024/7903972)
Supplement: Supplementary Materials — Table A1: Pearson's Correlation Coefficient for the associations between the single SEP variables (n = 2,716). Table A2: point-biserial correlation coefficient for the association with adolescent's obesity. [file 7903972.f1.zip › Supplementary material_ Table A1.docx]

**Supplementary Materials**

Table A1: Pearson's Correlation Coefficient for the associations between the single SEP–variables (n= 2,716).

|  |  | **Education** | **Occupational status** | **Household income** | **SEP–index** |
| --- | --- | --- | --- | --- | --- |
| **Education** | Coef. | 1.00 |  |  |  |
|  | p |  |  |  |  |
| **Occupational status** | Coef. | 0.53 | 1.00 |  |  |
|  | p | <0.001 |  |  |  |
| **Household income** | Coef. | 0.57 | 0.43 | 1.00 |  |
|  | p | <0.001 | <0.001 |  |  |
| **SEP–index** | Coef. | 0.85 | 0.76 | 0.85 | 1.00 |
|  | p | <0.001 | <0.001 | <0.001 |  |
