# Supplementary material for: Association between Socioeconomic Position of the Family and Adolescent Obesity in Germany—Analysis of the Mediating Role of Familial Determinants
Source: J Obes. 2024 Nov 5;2024:7903972. doi: 10.1155/2024/7903972 (PMC11557177; doi:10.1155/2024/7903972)
Supplement: Supplementary Materials — Table A1: Pearson's Correlation Coefficient for the associations between the single SEP variables (n = 2,716). Table A2: point-biserial correlation coefficient for the association with adolescent's obesity. [file 7903972.f1.zip › Supplementary material_ Table A2.docx]

**Supplementary Materials**

Table A2: Point–Biserial Correlation Coefficient for the association with adolescent’s obesity.

|  | **Male adolescents** | | **Female adolescents** | |
| --- | --- | --- | --- | --- |
|  | **Coef.** | **p** | **Coef.** | **p** |
| **Exposure** |  |  |  |  |
| Education | –0.10 | <0.001 | –0.10 | <0.001 |
| Occupational status | –0.07 | 0.009 | –0.10 | <0.001 |
| Household income | –0.08 | 0.002 | –0.12 | <0.001 |
| SEP–index | –0.11 | <0.001 | –0.14 | <0.001 |
| **Mediator** |  |  |  |  |
| Family cohesion | 0.02 | 0.667 | –0.02 | 0.483 |
| Parental stress | 0.03 | 0.227 | –0.02 | 0.530 |
| Parental overweight | 0.15 | <0.001 | 0.12 | <0.001 |
| Parental smoking | 0.10 | <0.001 | 0.10 | <0.001 |
| Parental sporting activity | –0.10 | <0.001 | –0.07 | 0.005 |
